# Unveiling the Silent Menace: A Case Report of Extrapulmonary Tuberculosis Masquerading as Neuroendocrine Tumor With a Brief Review of the Literature

**DOI:** 10.7759/cureus.69755

**Published:** 2024-09-19

**Authors:** Madhu Mathi, Swathy Moorthy, Lakshmi M, Bhaskar Emmanuel

**Affiliations:** 1 General Medicine, Sri Ramachandra Institute of Higher Education and Research, Chennai, IND; 2 Internal Medicine, Sri Ramachandra Institute of Higher Education and Research, Chennai, IND

**Keywords:** low saag ascites, chronic ascites, positron emission tomography-computed tomography in abdominal tuberculosis, extrapulmonary tuberculosis, neuroendocrine tumor

## Abstract

We present the case of a 59-year-old man from South India who presented with shortness of breath, abdominal distention, and decreased appetite. Initial laboratory investigations and imaging, including radiolabeled somatostatin positron emission tomography-computed tomography, were suggestive of neuroendocrine tumor (NET). However, following extensive workup and multiple biopsies over several weeks, the diagnosis was revised to extrapulmonary tuberculosis (EPTB). This case highlights the diagnostic challenge of distinguishing between NET and EPTB. We also review varying literature on the pathogenesis and similarities between these two conditions, discuss the role of laboratory investigations and imaging in identifying overlapping findings, and compare the diagnostic modalities used for NET and EPTB.

## Introduction

Tuberculosis (TB) remains a significant global health issue, ranking as the 13th leading cause of death and the second leading infectious cause of death after COVID-19, surpassing HIV/AIDS [1]. In 2021, approximately 1.6 million deaths were attributed to TB worldwide. According to the World Health Organization (WHO) statistics for India in 2021, the number of TB cases was estimated at 2.59 million, translating to an incidence rate of 188 per 100,000 population [2]. Globally, it is estimated that 10.6 million people contracted TB in 2021, including six million men, 3.4 million women, and 1.2 million children [3].

Extrapulmonary TB (EPTB), which affects organs and tissues beyond the lungs, constitutes a significant aspect of the disease. EPTB accounts for 10%-20% of all TB cases and can involve various sites, including the pleura, lymphatic system, skeletal and muscular tissues, gastrointestinal tract, and genitourinary system [4,5]. Despite the predominance of pulmonary TB, the extrapulmonary form is clinically important because of its diverse manifestations.

EPTB can present with symptoms that mimic other conditions, including malignancies. Neuroendocrine tumors (NETs), although rare, are malignancies originating from neuroendocrine cells and can sometimes be confused with EPTB because of their varied presentations [6]. The differentiation of NET and TB, particularly EPTB, can be confusing because of their overlapping clinical, biochemical, and pathological features. Therefore, we present a case that highlights various clinical manifestations and diagnostic challenges associated with EPTB.

## Case presentation

A 59-year-old man with known diabetes and hypertension presented to the outpatient department with complaints of shortness of breath, abdominal distension, and decreased appetite over the past three months. Initial physical examination revealed hepatomegaly extending 4 cm below the right costal margin with gross ascites. Visible dilated, nontortuous veins were seen around the umbilicus. Additionally, on auscultation, there was diminished air entry in approximately 30% of the right lung fields. A baseline blood investigation revealed anemia and elevated erythrocyte sedimentation rate, as seen in Table 1.

**Table 1:** Basic blood workup of the patient. dL: Deciliters; IU: International units

Laboratory parameter	Patient’s value	Reference range
Hemoglobin	11 g/dL	12-17 g/dL
Total leukocyte count	9,750/mm^3^	4,000–11,000/mm^3^
Platelet count	4.10/mm^3^	150,000–450,000/mm^3^
Erythrocyte sedimentation rate	24 mm/h	0–15 mm/h
Serum creatinine	1.1 mg/dL	0.8–1.3 mg/dL
Blood urea nitrogen	8 mg/dL	7.9–20.1 mg/dL
Total bilirubin	0.72 mg/dL	0.3–1.2 mg/dL
Serum aspartate transferase	25 IU/L	<50 IU/L
Serum alanine transferases	10 IU/L	<50 IU/L
Total protein	7.6 g/dL	6.6–8.3 g/dL
Serum albumin	2.8 g/dL	3.5–5.2 g/dL

**Figure 1:**
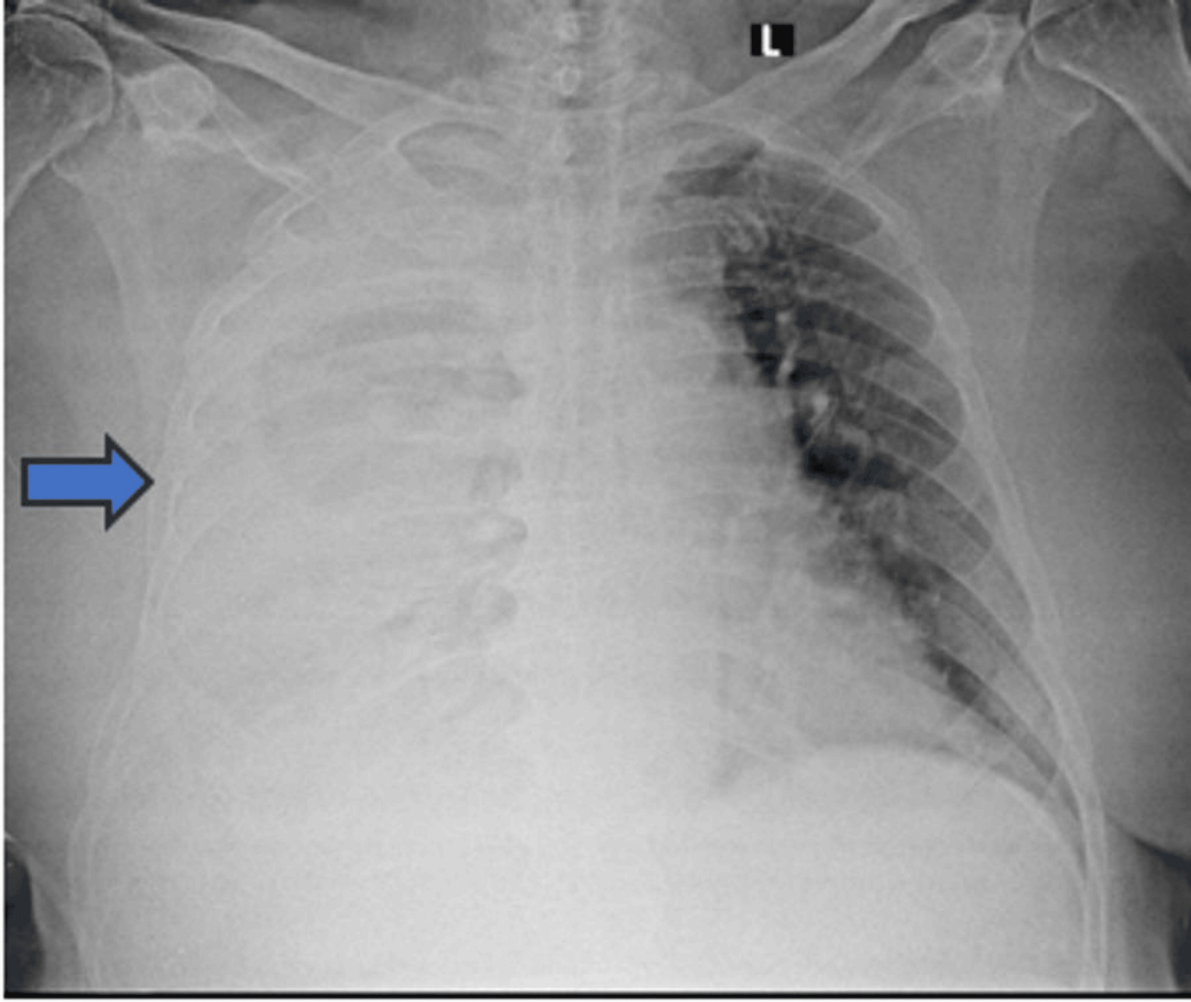
Radiograph of chest showing moderate pleural effusion (blue arrow).

The initial chest radiograph showed mild bilateral pleural effusion. During his hospital stay, the patient had desaturation and absent breath sounds in 50% of the right lung fields. A repeat chest X-ray revealed a moderate right pleural effusion (Figure 1).

Diagnostic thoracocentesis was done (i.e., pleural fluid tapping), and the sample was sent for biochemical, pathological, and microbiological analyses, which showed an exudative pattern (per Light’s criteria) [7]. Cell cytology revealed a monocytic-predominant cell count (Table 2) and an elevated pleural fluid adenosine deaminase (ADA) level of 64.5 International Units (IU)/L. However, a microbiological workup of the pleural fluid analysis, including acid-fast bacilli (AFB) smear, GeneXpert MTB (Cepheid, Sunnyvale, CA), and cultures, was found to be negative.

**Table 2:** Patient's Pleural fluid analysis report.

Laboratory parameter	Patient’s value	Reference range
Pleural fluid protein	4.9 g/dL	<3 g/dL
Pleural fluid lactate dehydrogenase	126 U/L	<40 U/L
Serum lactate dehydrogenase	144 U/L	300 U/L
WBC count in pleural fluid	838 cells/mm^3^	500–1,000 cells/mm^3^
Polymorphonuclear neutrophil cell count	153 cells/ mm^3^	
Mononuclear cell count	685 cells/ mm^3^	

**Figure 2:**
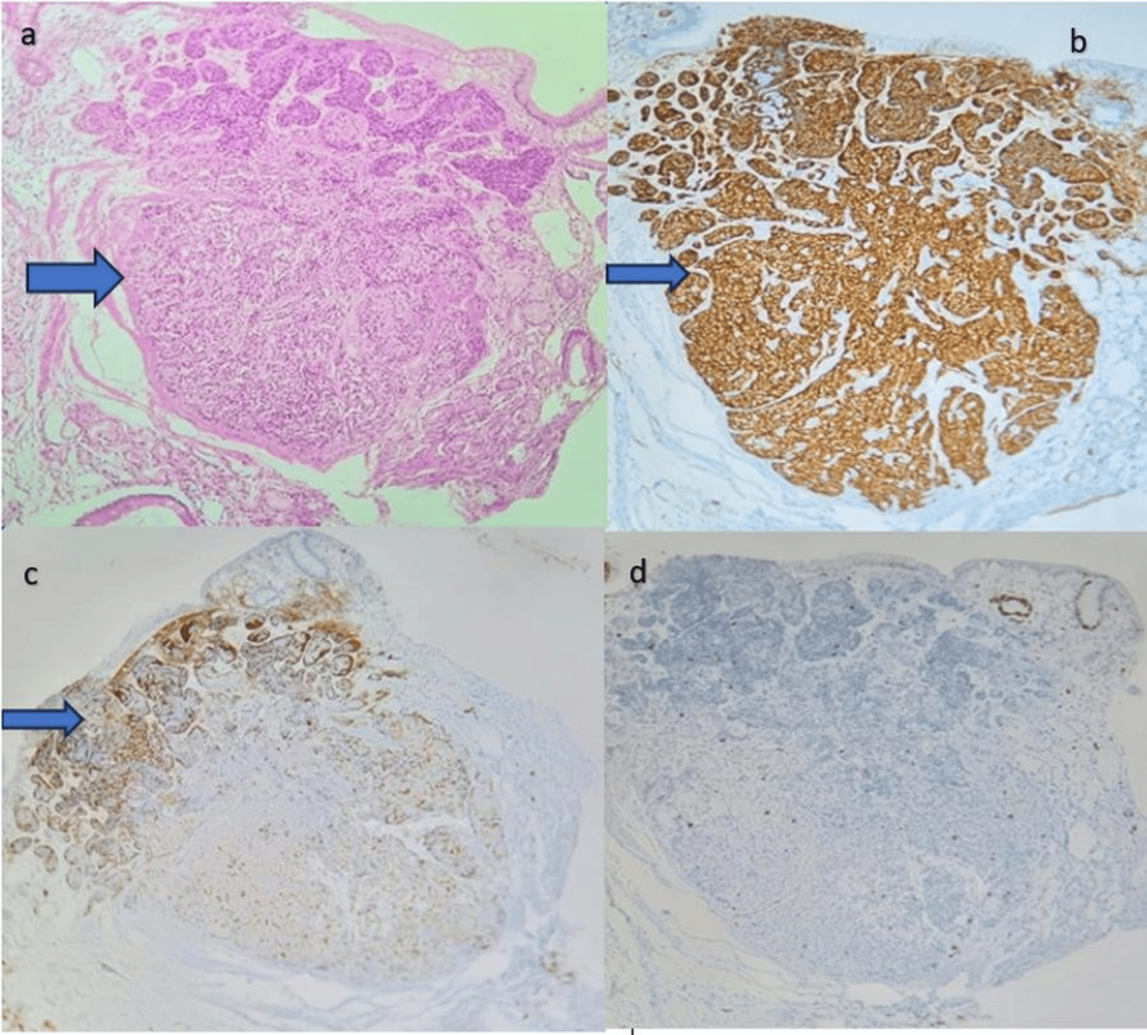
Immunohistochemistry indicating that the lesional cells showed positive uptake for synaptophysin and chromogranin. (a) Duodenal biopsy showing well-circumscribed neoplasm in the submucosa with nests of tumor cells; (b) immunohistochemistry for synaptophysin showing strong positivity in the tumor cells; (c) immunohistochemistry for chromogranin showing focal positivity in the tumor cells; (d) immunohistochemistry indicating a Ki-67 labeling index of 1% in the tumor cells, suggesting all markers for neuroendocrine tumor were positive.

Ultrasound of the abdomen showed hepatomegaly (18.4 cm), altered echotexture with surface nodularity, splenomegaly (13 cm), and moderate to gross ascites. Based on the ultrasound findings and clinical examination, our initial workup focused on liver pathology. Liver elastography (FibroScan, Echosens, Paris, France) showed a grade 1 fibrosis (elasticity of liver-shear wave elastography=7.5 kilopascals (kPa)) and grade 1 steatosis (ultrasound-guided attenuation parameter=245.4 dB/m). Upper gastrointestinal endoscopy was performed to assess for the varices as an indication of portal hypertension, which revealed antral erythema and a duodenal nodule. A biopsy of the duodenal nodule revealed a well-circumscribed neoplasm arranged in nests, with preserved villous architecture and no evidence of neuronal increased mitosis, which was positive for Ki-67 labeling index 1% in the tumor cells. Immunohistochemistry indicated that the lesional cells showed positive uptake for synaptophysin and chromogranin, suggesting NET (Figure 2).

Diagnostic paracentesis (ascitic fluid tapping) was done, and the sample was sent for microbiological, pathological, and biochemical analyses, which revealed a low serum-ascitic albumin gradient (SAAG) of 0.7 and a high protein ascites level of 2.8 g/dL (i.e., higher than 2.5 g/dL) (Table 3).

**Table 3:** Patient's ascitic fluid analysis report.

Laboratory parameter	Patient’s value	Reference range
Ascitic fluid protein	5.2 g/dL	<3 g/dL
Ascitic fluid albumin	2.1 g/dL	>1.1 g/dL
Serum protein	7.6 g/dL	6.6–8.3 g/dL
Serum albumin	2.8 g/dL	3.5–5.2 g/dL

**Figure 3:**
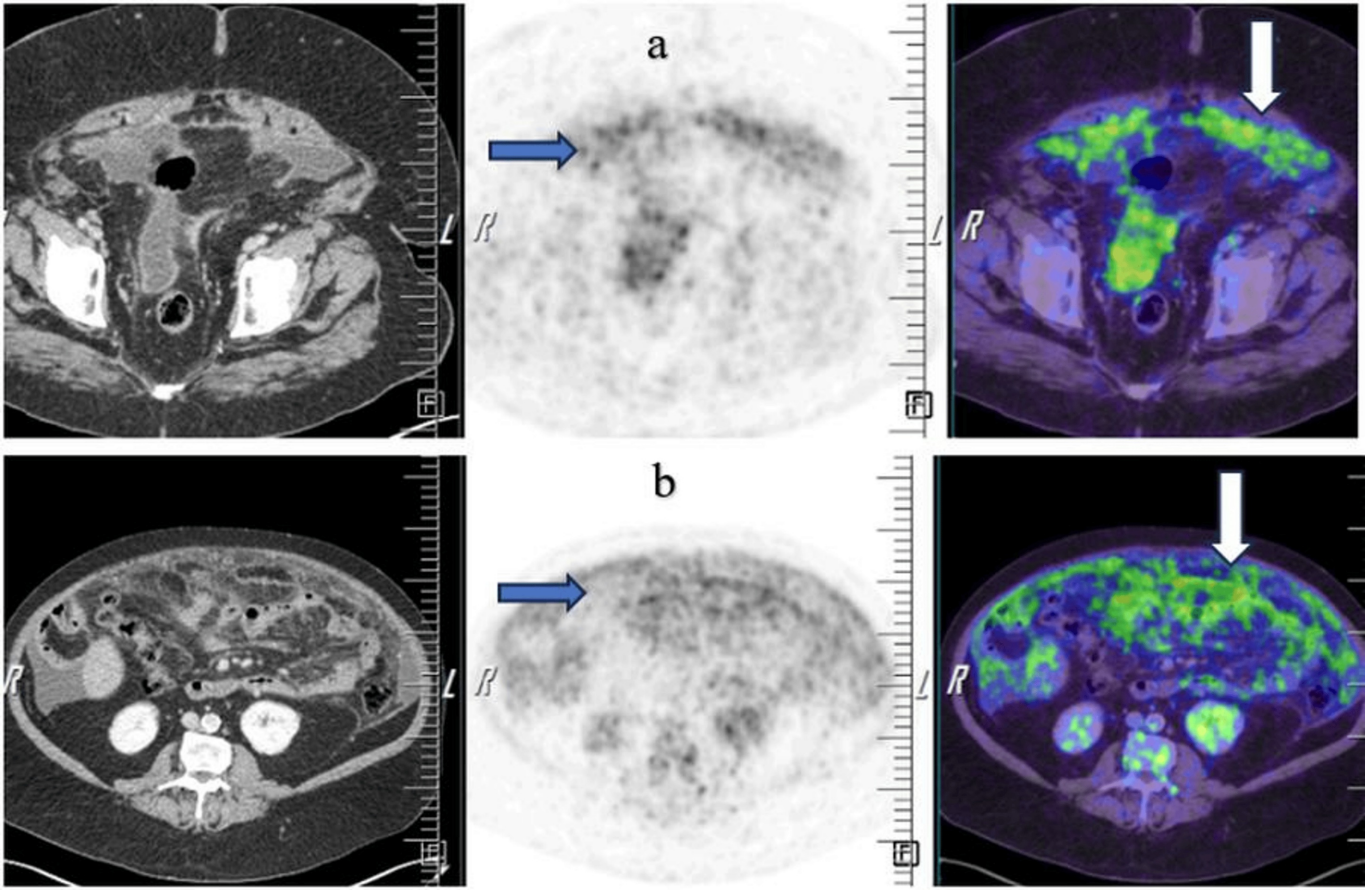
a and b: Positron emission tomography-computed tomography showing fluorodeoxyglucose (FDG)-avid and diffuse omental thickening. Diffuse mesenteric and omental fat stranding shows patchy, scattered areas of increased FDG activity.

Histopathology and ascitic workup indicated malignancy. Therefore, whole-body PET-CT was done, which showed fluorodeoxyglucose (FDG)-avid and diffuse omental thickening (up to 2.7 cm) with no omental nodules. There were also mild to moderate ascites with diffuse mesenteric and omental fat stranding showing patchy scattered areas of increased FDG activity and mildly enhanced peritoneal thickening seen in the lower abdomen and pelvis (Figure 3).

**Figure 4:**
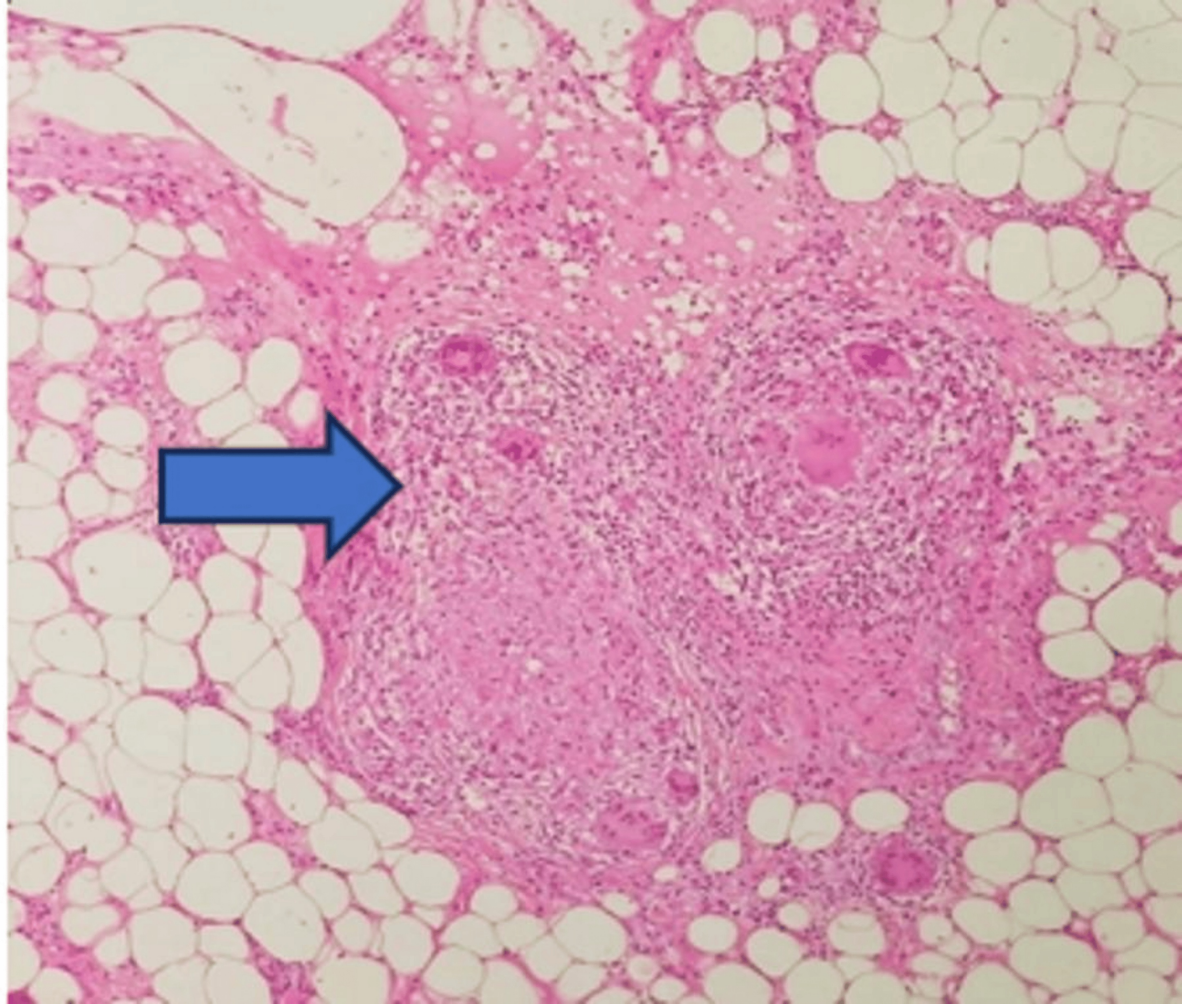
Omental biopsy revealing well-formed epithelioid granulomas with giant cell reaction.

A diagnostic laparoscopy was performed because of suspected malignancy, which revealed multiple omental adhesions. A biopsy of the omental tissue showed epithelioid granulomas with numerous Langhans-type multinucleated giant cells. A few areas showed caseous necrosis and lymphoid aggregates (Figure 4). An omental tissue sample was sent for GeneXpert MTB and culture. GeneXpert testing of the tissue detected Mycobacterium tuberculosis with indeterminate resistance to rifampicin, and the culture was positive for AFB. Consequently, the patient was started on antituberculous therapy (ATT).

A first-line line probe assay (LPA) was sent, as a cartridge-based nucleic acid amplification test showed indeterminate rifampicin resistance. The first-line LPA was found to be negative. The patient was continued on oral isoniazid 300 mg, oral rifampicin 450 mg, oral pyrazinamide 1,250 mg, and oral ethambutol 400 mg. Serial liver function tests showed increased total bilirubin levels reaching more than two times the upper limit of the normal range and aspartate transaminase and alanine aminotransferase levels more than three times the respective upper limits of the normal range. Therefore, the patient was changed to a modified ATT regimen including oral linezolid 600 mg, moxifloxacin 500 mg, and clofazimine 300 mg with oral ethambutol 400 mg.

On follow-up, a physical examination revealed decreased air entry in the right infrascapular region despite being on ATT. A repeat ultrasound of the thorax revealed a moderate pleural effusion on the right side (900-1,000 cc) with lung collapse. Therapeutic thoracocentesis was performed. Because of the reaccumulation of pleural fluid, incorporating an additional liver-sparing antituberculous drug was decided. Consequently, the patient was started on oral bedaquiline. The patient showed steady improvement over two months of follow-up and continued the modified ATT regimen.

## Discussion

EPTB can manifest in multiple ways depending on the affected organ(s). Common sites include the lymph nodes, pleura, bones, joints, genitourinary tract, central nervous system, and abdomen. Clinical presentations are often nonspecific, leading to diagnostic challenges. For example, TB lymphadenitis can mimic malignancies, whereas skeletal TB can mimic various bone disorders [8]. Early recognition is crucial to prevent complications and transmission. Key reasons for misdiagnosis of EPTB include nonspecific symptoms, mimicking other disease patterns, granulomatous inflammation, imaging similarities, systemic involvement, and biochemical and laboratory challenges. Because of these overlaps, a high index of suspicion and a comprehensive diagnostic approach, including clinical, radiological, and histopathological evaluation, is necessary to accurately distinguish between NETs and TB.

This case highlights how laboratory tests and imaging techniques commonly used to diagnose NETs can sometimes yield false positives in other conditions, particularly in granulomatous infections such as TB [8]. In our case report, the initial workup suggested a NET; however, both imaging and laboratory markers were misleading. Biochemical and investigative similarities do exist between both tuberculosis and NET because of their pathological mechanisms. Chromogranin A (CgA) is typically a marker for NETs and is not specific for TB. However, it can be elevated in various conditions besides NETs, such as renal failure, critical illness, and chronic inflammation such as TB. Although the DOTATATE PET scan is often used to detect NETs, it can also show positivity in chronic granulomatous diseases such as TB, leishmaniasis, and aspergillosis. This overlap is because of the high expression of membrane somatostatin receptors in activated macrophages, which can lead to diagnostic confusion with NETs [9]. Laboratory markers also can be misleading. For example, the sensitivity and specificity of AFB smear and culture in pleural and peritoneal fluid are relatively low compared to ADA [10,11]. Aggarwal et al. found that an ADA level of >65 IU/L had a sensitivity of 86% (95% CI: 0.61-0.96) and a specificity of 94% (95% CI: 0.80-0.99) in diagnosing TB compared to routine methods such as AFB smear [12].

**Figure 5:**
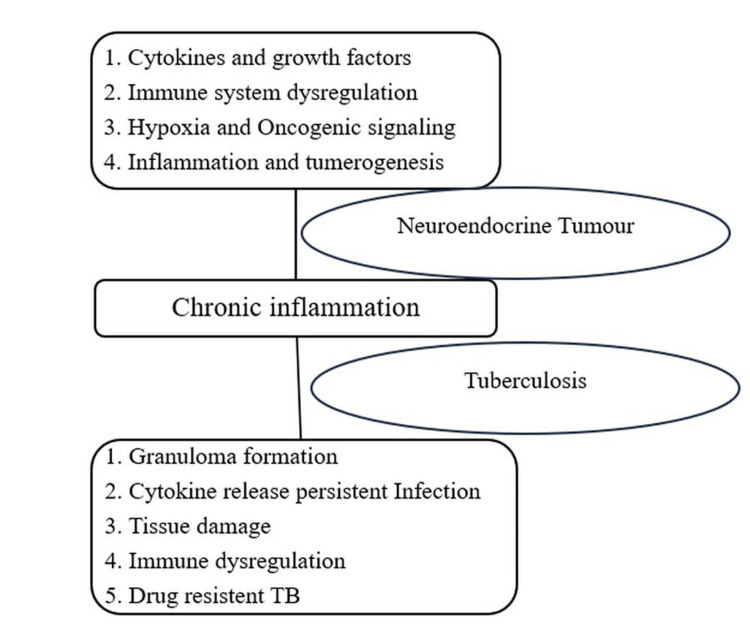
Role of chronic inflammation in neuroendocrine tumor and tuberculosis.

Based on various literature, chronic inflammation plays a major role in both the occurrence of NET and TB. Figure 5 explains the role of chronic inflammation in the occurrence of NET, where repeated exposure of tissues to inflammatory stimuli causes DNA damage, mutations, and epigenetic changes in oncogenic signaling pathways. All these factors result in uncontrolled cell growth, which is a hallmark of malignancy [13]. Additionally, dysregulation of inflammatory pathways can activate signaling pathways that promote tumorigenesis. For example, the nuclear factor kappa B pathway, a major player in inflammation, is associated with cancer development and progression, including NETs [14].

Figure 5 explains the role of chronic inflammation in TB. The immune system acts to control infection when M. tuberculosis enters the body. Often, macrophages can only ingest and cannot eliminate these bacteria, leading to the formation of granuloma. Granuloma is an organized structure of immune cells, including T-cells, fibroblasts, and macrophages, which is a hallmark of chronic inflammation in TB. Proinflammatory cytokines such as TNF-alpha and IL-1 are released by immune cells within granulomas, which then attract more immune cells and cause an inflammatory reaction, resulting in chronic inflammation, scarring, tissue damage, and systemic symptoms [15].

Therefore, there are significant diagnostic challenges in distinguishing between TB and NETs. Careful interpretation of radiological findings, in conjunction with clinical history and laboratory results, is crucial to avoid misdiagnoses.

## Conclusions

The coexistence of NET and pulmonary TB is rare. When both diseases occur simultaneously in an individual, diagnosis proves challenging, often requiring a multidisciplinary approach, as one condition may mask the other. Clinicians must remain vigilant to the possibility that two distinct pathological processes could coexist in the same patient, with higher success rates of diagnosis with strong suspicion, especially in atypical or prolonged clinical symptomatology. Other causes, such as drug resistance or paradoxical responses, which are more common during therapy, are usually unlikely to be missed in the diagnosis. Our case heralds one such scenario, which could have gotten carried away with the diagnosis of NET if further evaluation had not been proceeded with. A strong clinical suspicion with a thorough workup helps in narrowing down the differential diagnosis and salvaging the patient.

Chronic inflammation has an important role in the progression and outcome of NETs and TB. However, the role of chronic inflammation is very different in these conditions. In the case of NETs, chronic inflammation has been shown to cause tumors through the modulation of the tumor signaling pathway. In the case of TB, chronic inflammation is a part of innate host immunity against controlling the infection. However, this discussion is an open area of research that requires additional investigation. Future examination of the interplay between chronic inflammation, immune responses, and tumorigenesis may uncover interconnections between these conditions that would otherwise seem disparate.
